# Post-lesson supervisory dialogue and instructional adaptation in physical education: a qualitative multiple-case study

**DOI:** 10.3389/fspor.2026.1971342

**Published:** 2026-10-09

**Authors:** Haifa Jamhour

**Affiliations:** College of Physical Education and Sports Sciences, Palestine Technical University–Kadoorie, Ramallah, Palestine

**Keywords:** instructional adaptation, Palestine, physical education, professional agency, reflective practice, supervisory dialogue

## Abstract

**Introduction:**

This qualitative cross-case comparison examined three physical education supervisor–teacher dyads in Palestinian government schools to explore how post-lesson supervisory dialogue was associated with opportunities for reflection and how discussed ideas were subsequently taken up in short-term practice.

**Methods:**

Data comprised three audio-recorded dialogues, three follow-up teacher interviews, three follow-up supervisor interviews, and one classroom observation per teacher. Reflexive thematic analysis, complemented by a focused sequential dialogue analysis of contiguous conversational turns, was used to analyze the data.

**Results:**

Four themes were generated: affirmation as a relational entry point; variation in reflective participation associated with question design; movement from stated intention to reported and observed adaptation; and power dynamics expressed through evaluative framing, interactionally ambiguous agreement, and the delayed visibility of teacher agency. Across the three cases, open and context-sensitive questions were followed by more elaborated teacher explanations, whereas presuppositional questions and direct recommendations were more often followed by brief agreement. Follow-up interviews and observations showed direct adoption, adapted implementation, and selective non-adoption.

**Discussion:**

These case-specific patterns document short-term instructional adaptation and agreement-implementation divergence, but they do not establish causal effects, sustained change, or measurable effects on student learning.

## 1 Introduction

Educational supervision is increasingly conceptualized as developmental rather than limited to inspection, compliance, or evaluation. Gordon (1) describes supervisory dialogue as involving a professional interchange of ideas through questioning, giving descriptive feedback, reflecting, and collaborative problem-solving that enhances both professional judgments and shared responsibilities for improvement. In contrast, Goldhammer (2) viewed clinical supervision as providing a connection with classroom observations, analyses, and discussions after lessons to enhance instructional practice and reduce reliance upon administrative inspections.

Recent frameworks incorporate developmental and clinical supervision, reflective practice, and technology-supported approaches (3). However, supervisory feedback is not simply the transmission of information from an expert to a teacher. Dialogic feedback research conceptualizes feedback as an interaction in which participants interpret, negotiate, and make sense of information before deciding whether and how to act on it (4, 5). Feedback uptake relies upon more than the message of a suggestion itself. Recipience, affect, professional judgment, and opportunities for action all contribute to feedback uptake (6, 7).

In supervision, the same processes occur within an institutional asymmetry. Supervisors may provide problem frames and preferred alternatives, while teachers remain responsible for their own professional judgments regarding whether recommendations will be accepted as-is, modified, or ignored altogether (8, 9).

The aforementioned dynamics are especially relevant in physical education (PE)—when teachers immediately have to decide what movements to allow, what equipment to use, how to group students by motor skills, what level of difficulty to assign tasks, how much space is available, and which students to encourage participation from. Decisions like those referenced above require situated professional judgment rather than standardized responses (10).

The constraints-led perspective focuses on learner constraints, task constraints, and environmental constraints—all of which impact the types of instructional adaptations that could realistically be made during a lesson (11). While this study applies the constraints-led framework more narrowly to understand the contextual feasibility of instructional adaptations (i.e., grouping, difficulty level, space, equipment, safety), it is not being used as a holistic explanation of teacher agency or supervisor interactions.

Teacher agency is central to this research because feedback uptake does not end with verbal agreement. An ecological view of teacher agency sees teacher agency as something that emerges as an outcome of the interactions between professional knowledge and values and the resources, relationships, and institutional contexts that are available to teachers (12). Within supervisory dialogue, agency may become visible when teachers explain or qualify decisions, propose alternatives, modify recommendations, selectively adopt suggestions, or reject elements judged unsuitable. These expressions can be more or less visible when the person offering feedback also occupies an institutionally senior supervisory role.

The Palestinian context is especially relevant. The national policy emphasizes student-centered teaching, teacher and supervisor development, evaluation reform, educational quality, infrastructure, continuity of learning and responsiveness to school circumstances (13–15). Palestinian research is calling for a move away from “monitoring” to more “developmental,” “collaborative” and “professional support” types of supervision (16). It also distinguishes between “short-term teacher training” and “sustained professional development that empowers teachers through ongoing reflection, involvement in school decision-making processes, and continuing work-based learning” (17). However, workload, scheduling, logistical difficulties, and limited opportunities for application may limit professional learning (18).

Research on supervision and coaching emphasizes reflection, relational support, role clarity, and institutional conditions (19, 20). Feedback research adds a further concern: the analytical focus should extend beyond how feedback is delivered to how recipients make sense of it and whether it is subsequently used, modified, or not taken up (4, 6, 7). In PE, there remains limited evidence connecting recorded post-lesson dialogue with teachers’ later interpretations and observable short-term instructional decisions while also attending to contextual feasibility and supervisory authority (21, 22).

While in Palestine supervision has been framed as an effective way for schools to support their teachers, improve educational quality, and provide professional growth (13–15), developmental supervision can be impacted negatively due to factors such as workload, scheduling, logistical challenges, and school culture; limited time available for supervisors to carry out their work; teacher preferences regarding supervision; and little relationship between the supervisor’s role and classroom practice (16–18, 23). These same constraints are compounded when considering the immediacy of decision-making that is required in physical education classes related to issues of student participation, perceived ability differences among students, class organization and structure, physical space, availability and condition of equipment, and student/teacher safety. Therefore, it is not just whether supervision has benefits for education, but rather how each supervisory move affects the classroom environment on a daily basis; how educators perceive and act upon the supervisory efforts made toward them while operating within institutional constraints; and how proposed practices will be implemented, modified, or rejected based on local teaching conditions.

Against this background, the study addressed the following research questions:

1. What purposes and interactional patterns are evident in the three post-lesson supervisory dialogues?
2. How are supervisors’ questions, affirmations, feedback, and recommendations taken up across successive turns, and how do teacher voice, professional reasoning, agreement, and disagreement become visible within these exchanges?
3. How are student participation, differentiation, lesson organization, space, equipment, and other contextual constraints discussed across the three dyads?
4. How did teachers report implementing, modifying, selectively applying, or not implementing ideas discussed during the post-lesson supervisory dialogues?
5. What alignment or divergence was evident among the recorded dialogues, teacher and supervisor follow-up accounts, and observed instructional practices, particularly regarding teacher agency and implementation decisions?

## 2 Materials and methods

### 2.1 Analytical framework

Reflection is defined by Alexander (24) and Mercer (25), with Johansson (22) and Zhou et al. (26) adding reflection in terms of considering students’ responses and/or classroom conditions when making a decision about instructional practice. Teachers’ professional agency has been understood as their ability to identify and explain their instructional choices, raise questions, propose alternatives to those provided by others, modify those recommended by others, selectively agree to the recommendations made to them, and/or reject components they deem inappropriate in an institutionally asymmetrical supervisory relationship (8, 9, 12). Therefore, feedback uptake is conceptualized here as distinct from verbal agreement. Brief agreement is viewed as interactionally ambiguous unless subsequent data clarify both how the suggestion was interpreted and whether it was enacted.

The three analytical perspectives served distinct and complementary purposes. Dialogic theory was used to examine the structure and reciprocity of the supervisory exchange—whether successive turns opened space for explanation, inquiry, development of ideas, or reconsideration (24, 25). Professional agency was used to interpret teachers’ capacity to explain, qualify, modify, accept, or reject recommendations under supervisory authority (8, 9, 12). The constraints-led framework was used only to interpret the contextual feasibility of instructional adaptations in relation to learner, task, and environmental conditions (11). These perspectives were used as sensitizing lenses rather than competing causal theories; Table 1 maps them to the research questions, data sources, and analytic procedures.

**Table 1:** Integration of research questions, data sources, analytic procedures, and interpretive lenses.

Research question	Primary data sources	Analytic procedure	Interpretive contribution
RQ1: Purposes and interactional patterns	Recorded dialogues	Focused sequential dialogue analysis + reflexive thematic analysis	Dialogic theory: initiating moves, teacher uptake, follow-up, and closure
RQ2: Uptake of questions, feedback, and recommendations	Dialogues + teacher/supervisor interviews	Sequential comparison of successive turns; within-case thematic comparison	Dialogic theory + professional agency: visibility of reasoning, qualification, agreement, and disagreement
RQ3: Contextual constraints	Dialogues + interviews + observations	Reflexive thematic analysis + within/cross-case comparison	Constraints-led lens: learner, task, and environmental feasibility
RQ4: Implementation, modification, or non-adoption	Teacher interviews + observations, linked to prior dialogue	Case-level triangulation and implementation coding	Professional agency + contextual feasibility
RQ5: Alignment/divergence across sources	All four data sources	Within-case matrices followed by cross-case comparison and negative-case analysis	Distinguishes recorded interaction, retrospective interpretation, and observed short-term practice

The focused sequential procedure examined contiguous supervisor initiation → teacher response → supervisor uptake/redirect/closure sequences. Initial thematic coding was primarily inductive; the three theoretical perspectives were used as sensitizing lenses during interpretation rather than as a fixed deductive codebook.

### 2.2 Research design

The study employed a qualitative cross-case comparative design of three physical education supervisor-teacher dyads in the government schools of Palestine. Each dyad was treated as one analytic case, followed by cross-case comparison of convergent, divergent and limiting patterns (27).

The study examined how supervisors and teachers conceptualized instructional problems, discussed contextual constraints, suggested adaptations, and subsequently reported or demonstrated implementation. The findings provide a cross-sectional comparison rather than evidence of supervisory development over time since each dyad only completed one supervisory cycle.

### 2.3 Study setting and context

This research study occurred in West Bank government school districts run by the Palestinian Ministry of Education during the first term of the 2024–2025 school year. Each participant had completed the “For Sport for All” in Palestine program, which is also called the “Quality Sports” program locally. This program was funded through grants from the French Development Agency (Agence Française de Développement) and supported by a collaboration between the French Sports Federation and the Palestinian Ministry of Education.

A total of 436 participants were part of this program, including 40 central team physical education teachers; 360 new teachers who were provided training to implement the Quality Sports program; and 36 supervisors. In the three cases presented here, the supervisors were responsible for observing teacher lesson plans and leading the discussion that followed the lesson. Therefore, the interactions in these examples were asymmetrical due to their positions within an organizational structure rather than being peer-to-peer. The study did not document or record the formal performance evaluations of these supervisors/teachers to determine if there were consequences based on disagreements or participation. Thus, “evaluation framing,” used in this article, refers to both the supervisor’s position in the organizational structure and how they structured the interaction. It does not refer to documented appraisals or documented sanctions. A separate measure of the length and closeness of each prior supervisor–teacher relationship was not obtained. Trust and defensiveness are therefore based on what can be inferred from the recorded dialogue and accounts given after the fact and not based on assumed familiarity.

### 2.4 Participants and sampling

The sample consisted of six people: three PE teachers and three PE supervisors. The sampling unit was the whole supervisor–teacher dyad as opposed to an individual program participant. Using criterion sampling and then maximum-variation sampling, the researcher selected cases. The criteria for eligibility were completion of the “For Sport for All in Palestine (Quality Sports)” program, having a recorded post-lesson supervisory dialogue, obtaining written informed consent from each member of the dyad, and having all follow-up items used in this study (supervisor and teacher interviews and follow-up classroom observation). From eligible cases, three information-rich dyads were selected to maximize contrast in teacher and supervisor gender, professional experience, school type, governorate, and PE teaching conditions. The purpose was analytical contrast across cases, not statistical representativeness, and no claim is made that the selected dyads represent the full program population.

Table 2 presents the participants’ characteristics. Pseudonyms were used to protect their identities.

**Table 2:** Participant characteristics.

Dyad	Role	Pseudonym	Gender	Age	Professional experience	School context	Governorate	Dialogue duration
1	Teacher	T1	Female	32	8 years	Girls’ secondary school; outdoor concrete court; limited equipment	Ramallah and Al-Bireh	22 min
1	Supervisor	S1	Male	45	15 years	—	Ramallah and Al-Bireh	—
2	Teacher	T2	Male	27	4 years	Boys’ basic school; small playground; limited sports equipment	Hebron	35 min
2	Supervisor	S2	Female	50	20 years	—	Hebron	—
3	Teacher	T3	Female	38	12 years	Mixed primary school; indoor gym available	Nablus	28 min
3	Supervisor	S3	Male	42	10 years	—	Nablus	—

### 2.5 Data sources and collection

The data comprised three audio-recorded post-lesson supervisory dialogues, one per dyad; three follow-up teacher interviews; three follow-up supervisor interviews; and one follow-up classroom observation per teacher. Immediately conducted in a school setting, the dialogues focused on instructional goals, participation, organization, differentiation, resource use and potential accommodations. Follow-up interviews were conducted approximately three weeks later and lasted between 22 and 34 min. They examined how the educators understood the supervisory dialogue, made choices regarding implementation and adjustments, and viewed contextual limitations. The structured observation data included documentation of the way lessons were organized; ways students participated in class; groupings (formative and informal); ways the educator differentiated instruction; uses of physical and material space in the classroom; types of questions asked by the educator; ways students’ safety was ensured during lessons; and the degree to which the adaptations discussed during the supervisory dialogue were enacted.

### 2.6 Translation process

The primary author translated chosen Arabic excerpts into English, referencing the recordings to maintain meaning, tone, and engagement. A different bilingual researcher examined a sample with the original Arabic to assess lexical equivalence, semantic coherence, and pragmatic suitability. Disagreements about ambiguities were discussed until agreement was reached and notes were kept for expressions that depend on culture or context.

### 2.7 Follow-up interview protocol

Semi-structured teacher interviews explored interpretations of the supervisory dialogue, implementation decisions, modifications, professional agency, and contextual constraints. Supervisor interviews examined the purposes of questions and recommendations, interpretations of teacher responses, teacher agency, and PE-specific constraints. The full guides are provided in Supplementary Materials 1, 2.

### 2.8 Follow-up classroom observations

One follow-up lesson was observed for each teacher using structured field notes. Observations documented instructional organization, participation, contextual conditions, and evidence of direct, adapted, selective, or absent implementation. Immediately after each lesson, descriptive evidence was recorded along with links to the preceding supervisory dialogue and relevant contextual conditions. Because only one lesson was observed per teacher, the findings indicate short-term implementation rather than sustained practice, implementation fidelity, teaching quality, or student learning. Supplementary Material 3 presents the observation domains and adaptation codes.

### 2.9 Data analysis

Analysis used Braun and Clarke’s six-phase reflexive thematic analysis (39, 40) as the overarching strategy and complemented it with a focused sequential dialogue analysis of the three recorded supervisory exchanges. The approach to analyzing dialogue as a continuous process, using a sequential and focused format, was specific to the research project but does not imply that this process represented all aspects of conversation analysis. Instead, the intention behind the process was to document both the order and the take-up of the conversational moves within a piece of dialogue, which are often lost with thematic coding. Drawing on interactionist accounts of feedback dialogue (4), each transcript was analyzed through contiguous sequences of a supervisor’s opening move, the teacher’s response to that move, and then the supervisor’s uptake, probing, redirecting, or closing of the sequence. Using this type of approach enabled researchers to identify such issues as whether an open-ended question was responded to with reasoned argument; whether a recommendation was agreed upon briefly; and how the supervisor would expand or close the sequence.

The two procedures were integrated in four linked stages. First, all dialogue transcripts, teacher and supervisor interviews, and observation records were repeatedly read and semantically coded; initial coding was primarily inductive, while dialogic theory, professional agency, and the constraints-led framework functioned as sensitizing concepts rather than a fixed codebook. Second, focused sequential analysis was applied to each complete supervisory dialogue to generate sequence-level memos on initiating moves, teacher uptake, supervisory follow-up, problem framing, contextual explanation, and conversational closure. Third, these memos were brought back into reflexive thematic analysis and compared with interview and observation evidence within each dyad before cross-case comparison. Fourth, candidate themes were revised against divergent and negative cases. Thus, sequential analysis contributed evidence about how interaction unfolded turn by turn, whereas reflexive thematic analysis generated the broader patterns of meaning across data sources. Follow-up interviews and observations were used to examine later interpretation and implementation, not to infer teachers’ unspoken intentions retrospectively. Because reflexive thematic analysis does not treat coding agreement as a validity criterion, no inter-rater coefficient such as Cohen’s kappa was calculated.

### 2.10 Trustworthiness

Trustworthiness was established using Lincoln and Guba’s (28) criteria for credibility, transferability, dependability, and confirmability. Credibility was enhanced in a number of ways including continued engagement with the data and use of qualitative evidence obtained from interviews and observational studies, as well as through comparison of data both within and across individual case studies, the evaluation of contradictory evidence, through the use of direct quotes, and by conducting member checks. The preliminary study findings were shared with all three teachers who then provided affirmation regarding whether or not they felt that the findings represented their own experience. Transferability was also enhanced through the detail of context; dependability was maintained through documentation of transcriptions, coding, theme development, and cross-case decision making; and confirmability was enhanced through linking interpretations to relevant quotes, observations, and self-reflection.

### 2.11 Ethical considerations

Permission for this research was obtained by Palestine Technical University–Kadoorie to conduct the research at government schools. Prior to participating in the research, all teachers and school supervisors were provided with a document outlining the purpose of the study, that their participation would be voluntary, how data would be recorded, the use of quotation, what they could expect regarding privacy/confidentiality and their rights should they decide to withdraw prior to completion of the study. A signed informed consent document was required before any recording began. In order to maintain participant anonymity, all references to names and/or schools, students' classmates, colleagues, etc., have been removed from the study and replaced with pseudonyms or broader descriptions. All digital recordings have been recorded digitally on a secure device that is password protected. Only members of the research team may access these recordings/transcripts. The data will remain secure for at least 5 years post-publication prior to permanent deletion according to approved Data Management Procedures.

### 2.12 Researcher positionality and reflexivity

The author’s background in physical education supervision gave him a basic understanding of the context; however, it could have biased his reading of supervisory practices based on past experience and professional expectations. Therefore, the reflexive notes allowed the author to capture and modify initial readings/interpretations rather than just report what actions he took. Initially, one common analytical approach was to take a teacher’s short verbal agreement as an indication of their acceptance. When compared to both T2’s and T3’s subsequent written reports, as well as observational data, the author found later revisions in addition to selectively ignoring previous agreements, which led to the revised analysis (i.e., coding such responses as interactionally ambiguous agreement) and the removal of all statements concerning a teacher’s intentions at the time they agreed. In the same manner, affirming opening statements were not taken as an indicator of psychological safety in and of themselves; instead, interpretations were limited to the accounts reported by each teacher post-encounter and the amount of detail present in the recorded communication. All emerging themes were regularly cross-referenced back to transcriptions, audio files from encounters, written interviews, observational data, and evidence that either supported or contradicted the author’s developing interpretation. Finally, the author generated the Arabic-language versions of all transcripts and then translated some excerpts into English. A second bilingual researcher reviewed a random sample of those translations for both semantic and pragmatic equivalence.

## 3 Results

The four themes are: affirmation as an initial relational way into the dialogue; how questions and the ways in which they are asked vary with regard to the reflective participation of teachers; the progression from stated intentions to reported and observed adaptations in instruction; and the expression of power relations through evaluative framing, interactionally ambiguous agreements, and the delayed visibility of teacher agency. Evidence showed that concerns and decisions made by teachers about implementing changes after the dialogues were conducted were not always seen in the first round of dialogues.

### 3.1 Theme 1: affirmation as a relational entry point to reflection

When dyads included affirmation, supervisors began their response by acknowledging effective elements of the lesson prior to commenting on instructional issues. This opening was followed by a teacher explaining why she did what she did, and both participants agreed in a post-interview that it helped facilitate subsequent feedback. Therefore, in this case, the use of affirmation is interpreted as an initial relational way into the dialogue rather than as evidence that using affirmation promoted trust or created a psychologically safe environment.

This pattern was particularly evident in Dialogue 1:

Supervisor: “Well done. Today you laid a solid foundation for sport. You were great. All students participated.”

Supervisor: “Your lesson objectives were clear and achievable. The transitions between activities were smooth.”

Teacher: “Thank you. I tried to make sure students stayed active the whole time because they lose focus quickly.”

(Dialogue 1, lines 1–4)

The sequence shifted from affirming and providing descriptive feedback to T1 explaining how her teaching choices addressed students’ engagement.

T1’s follow-up interview supported this interpretation:

“Starting with positive comments made me relax. I was able to listen better to the rest of the feedback.”

(T1 follow-up interview, lines 12–14)

S1 retrospectively defined the opening affirmation as “intentional”: “If I had gone straight to the problem with the goals, she might have become defensive.” S1 reported that she validated the successful parts of the lesson prior to bringing up the aspect of how wide the goals were for discussion, whereas T1 said that the positive beginning allowed her to hear what S1 had to say about the issues after the opening. The alignment of the two retrospective accounts provides support for a particular case-by-case analysis of affirmation as a way into relating to each other; however, neither of the accounts provides objective measures or data showing a shift in T1’s psychological state.

#### 3.1.1 Affirmation, trust, and the power to speak

Affirmation continued to exist in the context of an asymmetrical institutional relationship. In the second interview with T1, she said: “The positive comments made me feel good, but I also knew the supervisor was watching everything.” (lines 18–21). In this example, affirmative exchange existed alongside increased participation by teachers, who were still aware of the authoritative presence of their supervisor. Therefore, based on the evidence provided, supportive interactions and awareness of supervision can coexist. However, it cannot be concluded that the hierarchical nature of the interaction was diminished due to the use of affirmation.

The supervisor’s use of direct recommendations in Dialogue 3 was distinct to Dyad 3; however, the supervisor did not provide any affirmative comments about the lesson. In fact, the supervisor started the dialogue with a comment suggesting ways that she believed could assist him in improving his lesson: “The students are at three different levels—strong ones, average ones, and weak ones. The courts can be separated in like categories.” (Dialogue 3, Lines 18–22).

In addition, T3’s follow-up interview provided additional support for interpreting the supervisor’s initial comments as being primarily evaluative. “I was a little defensive because I had no idea what you thought was good..” (T3 Follow-Up Interview, Lines 22–24). As previously mentioned, this is a *post hoc* participant account as opposed to actual evidence that he felt defensive due to a lack of affirmations. Nevertheless, it provides some insight into why there was relatively minimal elaboration by T3 during the recorded dialogue.

### 3.2 Theme 2: question design and variation in reflective participation

There was a notable difference in how teachers responded to open and context-specific questions versus presuppositional questions and/or direct recommendations. Open and context-specific questions were most likely to elicit elaborated responses from teachers, while presuppositional questions and direct recommendations elicited brief agreements or limited elaboration. These patterns are based on the data set of three cases. Therefore, they represent descriptive patterns of interaction rather than evidence that a specific type of question either promotes or restricts reflection.

#### 3.2.1 Open contextual questions and elaborated explanation

The clearest example of context-sensitive questioning appeared in Dialogue 2:

Supervisor: “How did you reach this level with the students and the very small field space? What challenges did you face?”

Teacher: “Despite the small area of the field, we used to play in small teams in many classes until the students were able to hold handball and play with it at this level.”

Supervisor: “Did you feel a difference when applying the strategy, with a change from the rest of the traditional classes?”

(Dialogue 2, lines 1–3)

The supervisor’s open question was followed by T2 explaining how limited space affected his lesson plans and how he used a small-group activity. As such, the sequence of events in the recording offers direct evidence of contextual explanation after being invited to explain; the follow-up interview indicates that T2 retrospectively thought the questions were polite.

T2’s follow-up interview clarified why the question encouraged elaboration:

“It showed the supervisor understood my constraints. He didn’t just say the field is small. He asked how I managed it. That felt respectful.”

(T2 follow-up interview, lines 28–30)

S2 explained that she also asked the first two questions because she wanted to know what adjustments T2 made during the lessons before making her own suggestions. S2 said “real” conditions are important when thinking about what students reflect on. Thus, it can be concluded from both the dialogue and subsequent interviews that contextual inquiry presented opportunities for T2 to think through or articulate reasons why he adapted his teaching; the questions themselves did not cause T2 to reason about why he adapted.

#### 3.2.2 Self-evaluation and follow-up questions: guided reconsideration

A more guided form of reflection appeared in Dialogue 1:

Supervisor: “Did you feel there was a problem with student performance?”

Teacher: “Yes, but when the idea reached the students, I placed the cones.”

Supervisor: “Do you think the wide goal helps solve this problem?”

(Dialogue 1, lines 5–8)

The sequence transitioned from T1’s assessment of the students’ performance and how she used cones to an exploration of possible alternative arrangements in terms of the breadth of goals. Since T1 did not provide further detail regarding her last statement, data collected during interviews and observations were necessary to see if this movement toward alternative arrangements resulted in action.

#### 3.2.3 Presuppositional questions, direct recommendations, and brief agreement

A more directive pattern appeared in Dialogue 2:

Supervisor: “Why didn’t you put the students in a discussion for a minute to discuss what happened in each level of problems the students faced while playing?”

Teacher: “I will work on that next time.”

(Dialogue 2, lines 13–14)

Grammatically phrased as a question, the movement implied that some form of student conversation would be a part of this process. Furthermore, this move was also implicit in presenting a preferred method. When interviewed, T2 stated, “I will work on that next time,” which is very similar to simply stating an intention. There appears to be little negotiation at that point. However, no other reasons are given for T2 choosing such a short response.

T2 later reflected:

“That question was harder. I didn’t have a good reason. I just hadn’t thought of it.”

(T2 follow-up interview, lines 45–47)

S2 construed T2’s “I will work on that next time” as an affirmation of the recommendations, whereas T2’s subsequent explanation and practice revealed differences; he implemented a type of class discussion but not the separate pre-activity (warm-up) recommendation. Therefore, it is understandable that his initial agreement was viewed as interactionally ambiguous instead of being directly indicative of complete acceptance.

A comparable pattern appeared in Dialogue 3:

Supervisor: “The students have three levels—strong, average, and weak—and the courts should be divided accordingly.”

Teacher: “I agree with the supervisor about dividing the courts by student levels to increase competitiveness.”

(Dialogue 3, lines 18–22)

T3 had a preferable differentiation option from the supervisor, and there was no specific invitation for T3 to evaluate options prior to accepting it in the recording. T3 indicated that the strategy would enhance competition but did not discuss space issues or labeling issues with the students during their dialogue. Those were the only concerns raised during the follow-up interview and when implementing the strategy.

T3’s later interview clarified the reason for her brief initial response:

“Mostly the supervisor told me what to do. I didn’t mind because I agreed. But I didn’t have as much opportunity to reflect as I might have liked.”

(T3 follow-up interview, lines 35–37)

In the follow-up to the recorded supervision conversation (the second interview), S3 stated that she understood T3’s initial agreement as acceptance of differentiation. Additionally, she felt that contextual modifications were acceptable if they impacted the safety of students or met their instructional needs. She also stated that she supported T3’s decision to replace “strong,” “average,” and “weak” with “beginner,” “intermediate,” and “advanced.” Her explanation during this later interview shows that S3 agreed on the idea of differentiation but did not feel that the details of how it would be implemented were settled during the recorded conversation.

The key here is that there are different types of evidence in the data. The recordings represent what was said by each person in a specific sequence of conversations. However, the follow-up interviews provide evidence of participants’ subsequent interpretations regarding their actions and concerns about implementing the ideas generated during the recorded supervisory exchanges. Therefore, some aspects of reasoning may have appeared for the first time in the follow-up interviews; thus, it is not assumed that all reasoning occurred at the same level or with intentional withholding at the same time.

### 3.3 Theme 3: From stated intention to reported and observed instructional adaptation

Follow-up teacher interviews and classroom observations were compared with the recommendations and stated intentions recorded in each supervisory dialogue. These comparisons documented alignment, modification, and non-adoption across sources; they do not demonstrate that the supervisory dialogue caused the later instructional practices.

#### 3.3.1 Dyad 1: direct adoption of goal adjustment

In the follow-up interview, T1 reported:

“Yes, I adjusted the goal width in my next lesson. I used smaller goals and kept the cones as markers.”

(T1 follow-up interview, lines 52–54)

Data from the subsequent observation was consistent with what T1 reported in her interview: The cones were used to mark the boundaries of the game and where the balls would be placed at the goal (narrower than before), and students were engaged as they worked in small groups. Overall, these three types of data are consistent with an initial adaptation to the new approach; however, they provide no evidence that it will continue into the future or improve the learning for students.

#### 3.3.2 Dyad 2: adapted implementation and selective non-adoption

In the follow-up interview, T2 reported:

“Yes, I tried it in the next lesson. I gave students one minute to discuss problems they faced during the game.”

(T2 follow-up interview, lines 58–60)

T2 implemented the student-discussion idea in modified form by adding the question, “What made it difficult to score?” During the observed one-minute discussion, students identified difficulties involving defenders, space, positioning, and passing, and T2 asked teams to identify strategies they considered effective. In contrast, the separate warm-up recommendation was not adopted. The combined dialogue, interview, and observation evidence therefore supports adapted implementation of one recommendation and selective non-adoption of another without requiring an inference about T2’s intention during his earlier brief agreement.

#### 3.3.3 Dyad 3: adapted implementation of differentiation

In the follow-up interview, T3 reported:

“Yes, I divided the courts by skill level. But I made some changes. I didn’t want the weaker students to feel labeled.”

(T3 follow-up interview, lines 65–67)

The results from the second session (T3) showed an example of a teacher adapting the concept of “differentiation” to what she called “zones,” which were labeled “beginner,” “intermediate,” and “advanced.” However, this time she did not label students; instead, she organized them into three zones with corresponding levels of activity. She also provided differentiated support. This result supported her claim that although the principle of “differentiation” was retained, it was reframed in terms of reducing the perception of labeling through modifications in the wording of the tasks and the structure of the lesson. Although only one lesson was evaluated, it demonstrated evidence of short-term adaptation for the purpose of implementing “differentiation.”

### 3.4 Theme 4: supervisory authority and the visibility of teacher agency

Institutional power dynamics were present throughout all three dialogues with regard to how supervisors interacted with their teachers. The ways in which supervisors defined the roles of observers, initiators of dialogue topics, framers of instructional issues/problems, and providers of alternative solutions illustrate how they maintained institutional authority. On the other hand, teacher agency emerged as a means of expressing explanations and qualifications in response to supervisors’ questions and statements and continued to become increasingly apparent in later discussions regarding modifications made by teachers, along with selective adoption/non-adoption of suggestions offered by supervisors.

#### 3.4.1 Institutional authority and evaluative framing

The interactional evidence from direct observation of how these issues were addressed in dyadic interactions occurred through the actions of their supervisors, who would initiate discussion on an evaluative topic, identify a problem, and propose a solution. In Dyad 3, for example, the supervisor established skill difference as the basis for proposing level-based court division; in the recorded exchange, T3 agreed but stated concerns in the subsequent follow-up interview regarding the labels assigned: “I said yes because it seemed logical, but I did not agree with the terms..” (lines 40–42). Although this illustrates that there can be a disconnect between what occurs in the dialogue and what occurs in a post-dialogue interview, this does not indicate whether institutional authority affects T3’s willingness to express concern over the labels.

#### 3.4.2 Interactionally ambiguous agreement and agreement–implementation divergence

A pattern also emerged from Dyads 2 and 3 in terms of how they described a series of events that included both brief agreement in the dialogue followed by modifications that were either partially implemented or not implemented at all. While it cannot be determined why teachers responded as they did when agreeing, a teacher’s brief affirmative response may have represented acceptance, politeness, uncertainty, lack of time, reluctance to challenge their supervisor’s ideas, or perhaps a decision to commit without having thought through the details.

#### 3.4.3 The limits and delayed visibility of teacher agency

Teacher agency was more visible in some follow-up accounts and implementation decisions than in the recorded dialogue itself. This does not mean that teachers lacked agency during the dialogue or deliberately concealed disagreement. Rather, the three cases show that brief exchanges did not always make qualification, feasibility concerns, or later modifications observable to the supervisor. Supervisors may therefore risk over-interpreting brief agreement if they do not explicitly invite teachers to identify unsuitable elements, alternatives, or conditions for implementation.

#### 3.4.4 Supervisory authority and the construction of instructional problems

Supervisors frequently initiated and framed the instructional issues discussed: S1 directed attention to goal width, S2 raised student discussion while also inviting explanation of space constraints, and S3 framed ability grouping as a response to skill differences. Dyad 2 contained the clearest teacher-generated contextual explanation, but supervisors generally retained interactional control over topic initiation and closure. This pattern provided structure and expertise while also making some teacher qualifications more visible only in later interviews or implementation. Table 3 summarizes the cross-case evidence and retains the distinction between directly observed dialogue, retrospective accounts, and follow-up practice.

**Table 3:** Cross-case comparison of supervisory dialogue and implementation.

Analytical dimension	Dyad 1	Dyad 2	Dyad 3
Case characteristics	T1: 8 years; S1: 15 years; girls’ secondary school; outdoor court; 22 min	T2: 4 years; S2: 20 years; boys’ basic school; small playground; 35 min	T3: 12 years; S3: 10 years; mixed primary school; indoor gym; 28 min
Supervisory approach	Affirmation, descriptive feedback, and guided self-evaluation	Contextual inquiry followed by directive questioning	Primarily direct recommendations
Teacher participation	Elaborated reasoning about participation, attention, cones, and goal width	Contextual explanation followed by brief agreement; disagreement with the warm-up recommendation was not expressed	Brief agreement with limited elaboration; concern about labels was not expressed
Follow-up evidence	Narrower goals and retained cones were reported and observed	Student discussion was modified and implemented; the separate warm-up recommendation was not adopted	Differentiation was implemented with revised labels and modified tasks
Teacher–supervisor alignment	Strong alignment regarding the recommendation and implementation	Partial alignment because S2 was unaware that T2 rejected the warm-up recommendation	Partial alignment because T3 independently modified the proposed labels
Agency and power	Guided questioning coincided with more elaborated teacher explanation	Selective agency became clearer during implementation	Modificatory agency became more visible after the supervisor-led dialogue
Implementation pattern	Direct adoption	Adapted implementation and selective non-adoption	Adapted implementation

The supervisors asked 14 questions in Dyad 1, 17 in Dyad 2, and 11 in Dyad 3. The categories describe practices observed during a single follow-up lesson and do not demonstrate sustained change or causal effects.

### 3.5 Cross-case differences

Teacher experience showed no consistent relationship with elaborative participation across these three cases. T1 provided detailed reasoning in a dialogue containing affirmation and guided inquiry; T3 provided limited elaboration in a more recommendation-oriented exchange; and T2 shifted from contextual explanation to brief agreement as the dialogue became more directive. School conditions also differed across implementation: T1 adjusted goal width on an outdoor court, T2 used small-group discussion in a restricted playground, and T3 created differentiated zones in an indoor gym. These are descriptive cross-case contrasts only; with three dyads, the study cannot estimate the independent influence of supervisory approach, experience, or context.

### 3.6 Alternative interpretations and negative-case analysis

Brief agreement had different meanings across the three cases because later evidence showed direct adoption, adaptation, and selective non-adoption. Professional reasoning sometimes became more visible during implementation than during the original dialogue. Ability grouping may support differentiated challenge but can also risk labeling or unequal participation, and one follow-up observation cannot establish students’ longer-term experience. Brief agreement may reflect acceptance, politeness, time pressure, uncertainty, reluctance to disagree, or an unresolved decision. The analysis therefore focuses on the observable divergence among verbal responses, retrospective accounts, and implementation rather than inferring internal intentions.

## 4 Discussion

Across these three cases, brief agreement during supervisory dialogue was not a reliable indicator of later feedback uptake. This finding is consistent with dialogic feedback scholarship, which treats feedback as an interpretive and interactional process rather than as one-way information delivery (4, 5), and with recipience research emphasizing the recipient’s role in making sense of feedback, judging its applicability, and taking action (6, 7). The present evidence extends that concern into PE supervision by comparing recorded dialogue with short-term follow-up accounts and practice. Given the three-case, single-cycle design, however, the study does not claim that question wording or supervisory authority caused the subsequent implementation decisions.

### 4.1 Affirmation, trust, and the conditions for reflection

The use of affirmation is consistent with developmental supervision and coaching approaches that emphasize relational support, reflective practice, collaboration, and evidence-informed feedback (1, 29–33). T1’s account also resembles feedback literature showing that affective and relational conditions can shape how recipients engage with feedback (6, 7). In this study, however, affirmation should be interpreted cautiously: one dyad reported it as helpful, but the design does not establish a general effect on trust or psychological safety. Trust is therefore better inferred from opportunities to explain, question, qualify, and disagree than from politeness or agreement alone.

### 4.2 Questioning does not automatically produce reflection

The results confirm that the manner of questioning should be carefully designed without suggesting that the act of questioning causes teachers to reflect. In this study, open-ended or contextual questions prompted longer answers than did prescriptive questions or directives. This finding is consistent with previous dialogical research on educational dialogue and strategies for supervisory conversations (22, 34–36), as well as previous literature on feedback (5)—both of which emphasize the importance of providing participants with opportunities for engagement and continued thinking over time. From a feedback perspective, the difference in questioning and directive formulations can also be understood in terms of the creation of understanding. While questions allow participants to determine how relevant the feedback is, directive formulations tend to create a quicker resolution to what has been said rather than allowing participants to continue exploring their own understanding (4).

Therefore, it is not the number of questions asked but rather the kinds of interactions those questions enable. Questions that ask participants to consider alternative perspectives, explain their rationale, and evaluate whether proposed solutions are feasible provide more opportunities for participants to demonstrate their reasoning processes. Conversely, when questions prompt an anticipated response, they may facilitate a conversational conclusion. Research on dialogical supervision and co-coaching also stresses the value of establishing clear roles among participants (37), having adequate time for conversations (20), and minimizing the evaluative stakes within which the conversations take place (38). Therefore, although prior research provides evidence supporting the plausibility of this study’s interpretation, the three dyadic data sets gathered here cannot establish a causal relationship regarding which supervisory action would lead to reflective behavior in other contexts.

### 4.3 Power, teacher voice, and agreement–implementation divergence

Professional agency should be understood as situated rather than as a stable trait expressed only through overt disagreement. Ecological accounts emphasize that agency is achieved through the interaction of professional capacities with relationships, cultural expectations, and structural conditions (12). In the present cases, supervisors often framed problems and preferred alternatives, while teachers later adapted, qualified, or declined aspects of recommendations. The resulting agreement–implementation divergence does not show strategic compliance; it shows that verbal agreement alone was insufficient evidence of uptake. More dialogic supervisory practice would therefore make feasibility, alternatives, and the legitimacy of modification or non-adoption explicit topics of discussion**.**

### 4.4 Contextual constraints and responsive teaching

Responsive PE instruction is inseparable from the physical and organizational conditions in which it occurs. Space, equipment, group size, student heterogeneity, task organization, and safety were relevant to both the discussions and the later adaptations. The constraints-led framework helps classify these feasibility conditions (11): T1 altered a task constraint through goal width, T2 organized participation in response to limited space, and T3 adjusted task complexity in relation to student skill. The constraints-led framework is limited to interpreting contextual feasibility. It does not explain supervisory power, teacher identity, or professional agency, which are addressed through the dialogic and agency perspectives.

### 4.5 A conceptual heuristic for future research

Figure 1 summarizes the tentative relationships observed across the cases, linking supervisory moves and relational conditions with teacher participation, professional agency, contextual constraints, and implementation patterns. It is a heuristic derived from the findings rather than a tested causal model.

**Figure 1:**
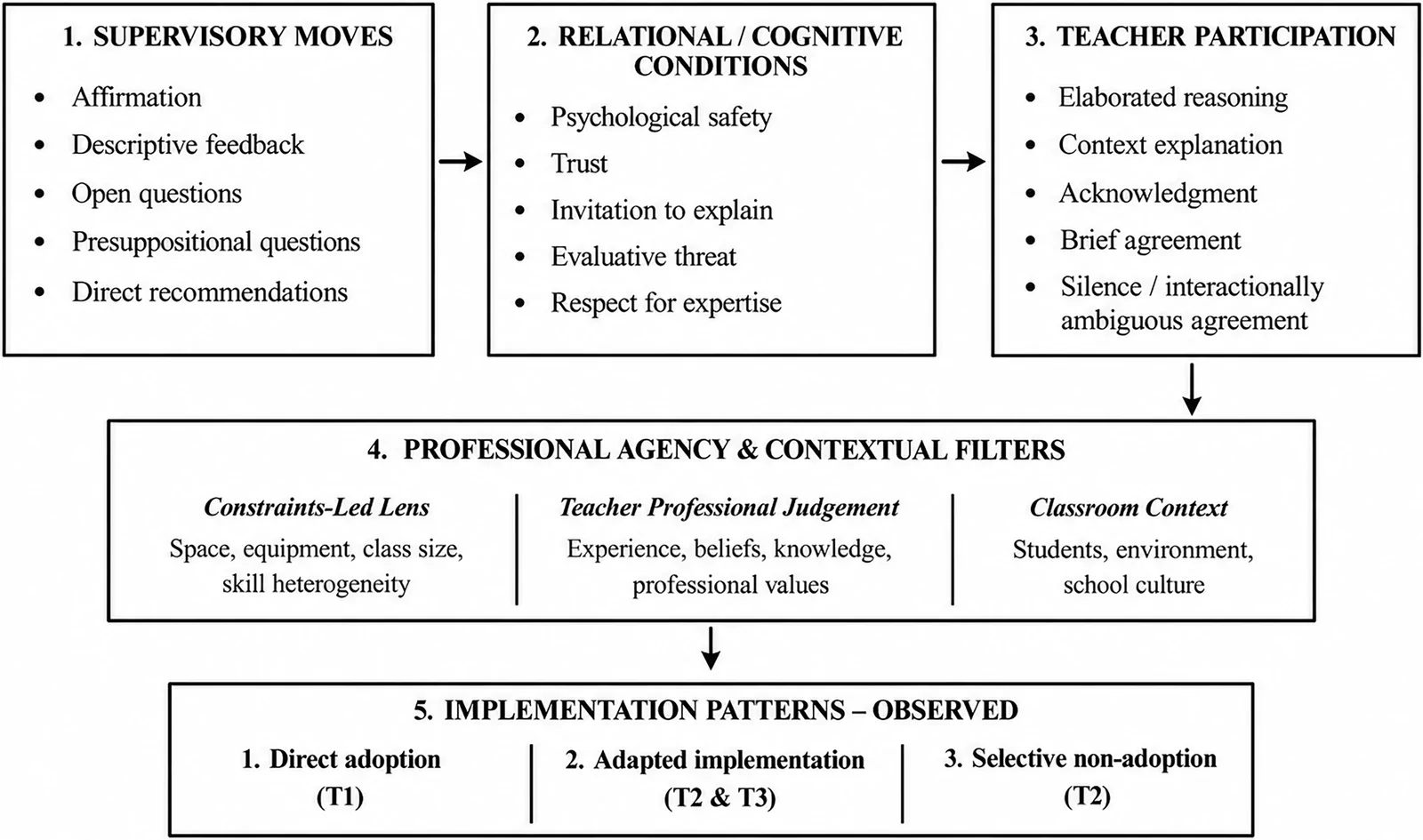
A heuristic of supervisory dialogue, professional agency, and instructional adaptation. Supervisory authority is represented as a contextual condition rather than a causal force. The arrows denote tentative, case-derived associations observed across three dyads and do not indicate causality or a validated model.

**BOX 1:** Illustrative supervisory dialogue prompts for physical education supervisors.

Instead of	Consider asking
“Why didn’t you use this strategy?”	“What alternatives did you consider?”
“You should divide the courts by ability.”	“How might you organize the space for students with different skill levels?”
“Did you feel there was a problem?”	“What challenges did you notice, and how did you respond?”
“I recommend changing this.”	“What would need to change to make this feasible?”
“Well done.”	“Your transitions kept students active. How did you organize them?”
“Do you agree with this recommendation?”	“Which parts of this recommendation fit your context, and which parts do not?”
“This is the main problem with the lesson.”	“What issue would you most like us to examine?”

The prompts presented in Box 1 are illustrative applications of the study findings rather than a validated supervisory instrument.” This provides the required in-text citation to Box 1.

Supervisors should also clarify that respectful disagreement, modification, and selective non-adoption are legitimate topics for professional discussion rather than evidence of unwillingness to improve.

### 4.6 Contributions to knowledge

This study contributes a micro-level, case-based analysis of supervisory dialogue in Palestinian physical education by connecting successive conversational turns with later teacher interpretations and short-term instructional practice. It highlights two tentative descriptive ideas: agreement–implementation divergence and the delayed visibility of teacher agency. These concepts draw attention to instances in which brief agreement did not reveal later modification or selective non-adoption, without attributing a specific intention to teachers at the moment of agreement. The study also shows that supportive interaction and institutional authority can coexist within the same supervisory encounter.

Methodologically, the study integrates reflexive thematic analysis with a focused sequential examination of dialogue. The sequential procedure preserves turn order and uptake, while reflexive thematic analysis identifies broader patterns across dialogues, interviews, and observations. This integration made it possible to distinguish what was directly observable in the interaction from what was reported retrospectively and what was observed later in practice. The proposed heuristic organizes these case-derived relationships for future testing and should not be interpreted as a validated model.

### 4.7 Limitations

This study involved only three supervisor–teacher dyads from one professional-development program, and each dyad contributed a single supervisory dialogue, one teacher interview, one supervisor interview, and one follow-up observation. The evidence is therefore case-specific, cross-sectional, and suitable for analytic rather than statistical generalization. The design cannot establish that any supervisory move caused a teacher response or later adaptation, and apparent cross-case differences may reflect unmeasured features of the teachers, supervisors, schools, or prior relationships.

It is recognized that retrospective accounts may be subject to potential influences from memory, socially desirable behavior, and self-presentation. Additionally, although reflective strategies were used to address the fact that the lead author supervised teachers (which could impact both how data were collected and analyzed), these strategies do not eliminate the possibility that the supervisors’ backgrounds impacted either the data collection or the analysis process. Also, the research study did not provide access to external administrative documentation related to the specific conversations examined to determine whether those conversations resulted in an evaluation process or whether disagreements had any type of consequence within the institution. Further, the study did not examine either the length or the quality of the relationships that previously existed between teachers and their supervisors. Therefore, when analyzing the authority to evaluate based upon position within an institution, the interactional frame used during conversation, and participant accounts, rather than documented sanctions or institutional policies, the results of this study can only be interpreted with respect to those areas.

Further, because no additional information was available regarding intentions not voiced during the conversation or which types of agreements reached were strategic, all responses are viewed as “interactionally ambiguous” rather than as examples of negotiated compliance. Finally, translation may have reduced some of the nuances of power and/or the meaning of interpersonal communication, and since no student interviews were conducted, limitations exist relative to making assertions regarding students’ experiences.

Since only one post-lesson observation occurred for each teacher involved in this study, evidence of implementation of practices was quite limited. While interview questions utilized specific probes that referenced specific parts of the dialogue examined during these lessons, other methods of collecting data that would allow the researchers to document implementation over time (i.e., repeated observations using audio- or video-recording and stimulated recall) were not utilized. Further, independent observational coding schemes were not developed or used in conjunction with other measures of student learning. As a result, while the data suggest that teachers at least briefly aligned themselves with their supervisors’ perspectives/expectations following one round of supervision, the data do not allow for conclusions regarding long-term changes in teaching practice, implementation fidelity of instructional practices, improvements in overall teaching effectiveness, or impacts on student engagement/participation levels, confidence, and motor skill acquisition.

## 5 Conclusion

Across these three cases, post-lesson supervisory dialogue showed four interrelated patterns: affirmation sometimes functioned as a perceived relational entry point; different question formulations were associated with different degrees of visible teacher elaboration; stated intentions did not always map directly onto later implementation; and institutional asymmetry was evident in how instructional problems and preferred alternatives were introduced. Teachers subsequently adopted, adapted, or selectively did not adopt recommendations in ways that were consistent with their professional judgment and teaching conditions. These findings should be read as case-specific descriptions of short-term supervisory interaction and uptake, not as evidence that particular supervisory moves caused reflection or instructional change.

The heuristic is therefore provisional. Research with larger and more diverse samples, repeated supervisory cycles, longitudinal observations, and stimulated-recall methods is needed to test whether the patterns identified here recur across settings and over time. Such work should also examine formal appraisal arrangements and prior supervisor–teacher relationships, because these may shape what teachers feel able to say during feedback conversations. Student-level outcomes would require separate measurement rather than inference from teacher or supervisor accounts.

For practice, the three cases suggest a cautious set of possibilities rather than prescriptive rules: supervisors can invite teachers to explain their reasoning before offering solutions, ask explicitly about contextual feasibility, distinguish developmental dialogue from formal evaluation where possible, and make modification or non-adoption discussable rather than treating agreement as the endpoint of feedback. Recommendations can be framed as proposals to be examined and adapted to the lesson context, while recognizing that the effectiveness of these practices requires evaluation beyond the present three cases.

## Data Availability

The raw data supporting the conclusions of this article will be made available by the authors, without undue reservation.
