# Supplementary material for: Post-lesson supervisory dialogue and instructional adaptation in physical education: a qualitative multiple-case study
Source: Front Sports Act Living. 2026 Oct 9;8:1971342. doi: 10.3389/fspor.2026.1971342 (PMC13690966; doi:10.3389/fspor.2026.1971342)
Supplement: Supplementary file 1 [file Supplementaryfile1.docx]

**Supplementary Material**

**Post-Lesson Supervisory Dialogue and Instructional Adaptation in Physical Education: A Qualitative Multiple-Case Study**

This supplementary file contains the teacher follow-up interview guide, the supervisor follow-up interview guide, and the classroom observation protocol used in the study.

**Supplementary Material S1. Follow-Up Semi-Structured Interview Guide for Physical Education Teachers**

**Timing:** Approximately three weeks after the post-lesson supervisory dialogue
**Duration:** 25–35 minutes
**Participants:** Three physical education teachers

**Opening Statement**

Thank you for participating. This interview explores how you interpreted the post-lesson supervisory dialogue, whether you attempted the ideas discussed, and what supported or constrained their implementation. Participation is voluntary, your responses are confidential, and you may stop at any time. With your permission, the interview will be audio-recorded.

**Interview Questions**

**1. What parts of the post-lesson supervisory dialogue do you remember most clearly, and why?**
**Probes:** Did any comment or question make you reconsider the lesson? How did you feel during the discussion?

**2. What changes, if any, did you make after the dialogue?**
**Probes:** In which lesson did you attempt them? Were they implemented as discussed, modified, postponed, or not implemented? Why?

**3. Which questions or recommendations were most useful, difficult, or unsuitable to apply?**
**Probes:** How did they relate to students’ needs, lesson planning, grouping, questioning, or activity organization?

**4. How did the supervisor’s questions influence your thinking?**
**Probes:** Which questions encouraged reflection or explanation? Did any feel evaluative, critical, or directive?

**5. Did you feel able to disagree with, question, or modify the supervisor’s recommendations? Why or why not?**
**Probes:** Did the dialogue feel collaborative or evaluative? Did you accept any recommendation without expressing concerns?

**6. What happened when you attempted the proposed change?**
**Probes:** How did students respond? What changes did you observe in participation, engagement, confidence, organization, or performance? Did any difficulty arise?

**7. During the dialogue, you made the following statement. What did it mean to you at that time?**

**T1:** “Yes, but when the idea reached the students, I placed the cones.”

**T2:** “I will work on that next time.”

**T3:** “I agree with the supervisor about dividing the courts by student levels to increase competitiveness.”

**Probes:** Did you intend to implement or modify the recommendation? Did you have concerns? Did you feel able to express a different view?

**8. How did contextual conditions affect implementation?**
**Probes:** Consider space, equipment, class size, students’ skill levels, lesson duration, school expectations, and safety.

**9. Did the dialogue influence how you evaluate, plan, or adapt your teaching? What additional support would have helped?**
**Probes:** Consider equipment, time, demonstration lessons, follow-up visits, peer mentoring, collaborative planning, and additional training.

**10. How could future post-lesson supervisory dialogues better support physical education teachers?**
**Probes:** Consider question design, teacher participation, timing, follow-up procedures, and conditions specific to physical education.

**Closing Question**

Is there anything else you would like to add about the dialogue, your instructional decisions, or the implementation of the proposed changes?

**Closing Statement**

Thank you for sharing your experience. Your responses will be analyzed alongside the post-lesson supervisory dialogue and follow-up observation data to examine how proposed adaptations were interpreted, implemented, modified, selectively applied, or declined.

**Supplementary Material S2. Follow-Up Semi-Structured Interview Guide for Physical Education Supervisors**

**Timing:** Approximately three weeks after the post-lesson supervisory dialogue
**Duration:** Approximately 25–35 minutes
**Participants:** Three physical education supervisors

**Opening Statement**

Thank you for participating. This interview explores your interpretation of the post-lesson supervisory dialogue, the reasoning behind your questions and recommendations, your interpretation of the teacher’s responses, and your consideration of the school context. Participation is voluntary, responses are confidential, and you may stop the interview at any time. With your permission, the interview will be audio-recorded.

**Interview Questions**

1. **What were your main goals during the post-lesson supervisory dialogue?**
   *Probes:* Were you seeking to evaluate, support, guide, or collaboratively address an instructional problem?
2. **How did you decide which aspects of the lesson to discuss?**
   *Probes:* Consider student participation, lesson organization, differentiation, space, equipment, and safety.
3. **Why did you use the particular questions, feedback, or recommendations evident in the dialogue?**
   *Probes:* Were you inviting teacher explanation, encouraging self-evaluation, or proposing a preferred alternative?
4. **How did you interpret the teacher’s responses during the dialogue?**
   *Probes:* How did you understand brief agreement, hesitation, explanation, or apparent acceptance?
5. **Did you feel that the teacher could disagree with, question, or modify your recommendations? Why?**
   *Probes:* Did you intentionally invite alternative views or concerns?
6. **How did you balance your professional authority with the teacher’s professional judgement and autonomy?**
   *Probes:* Were some recommendations presented as necessary while others were open to adaptation?
7. **How did contextual conditions influence your recommendations?**
   *Probes:* Consider space, equipment, lesson time, class size, student skill differences, and safety.
8. **Did you expect the teacher to implement the recommendation exactly as discussed or adapt it to the lesson context?**
   *Probes:* Which elements did you regard as essential, optional, or open to modification?
9. **Looking back, would you change any part of your questioning, feedback, or recommendations?**
   *Probes:* Consider question wording, teacher participation, timing, clarification, and follow-up.
10. **What conditions would make post-lesson supervisory dialogue more developmental and useful for physical education teachers?**
    *Probes:* Consider supervisor training, visit frequency, trust, time, resources, and separation from formal evaluation.

**Closing Question**

Is there anything else you would like to add about the dialogue, your supervisory approach, or the teacher’s subsequent instructional adaptation?

**Closing Statement**

Thank you for sharing your perspective. Your responses will be analyzed alongside the recorded supervisory dialogue, teacher interview, and classroom-observation evidence to examine alignment and divergence among supervisors’ intentions, teachers’ interpretations, and observed practice.

**Supplementary Material S3. Follow-Up Classroom Observation Protocol**

The protocol documented lesson organization, student participation, grouping and differentiation, use of space and equipment, task modification, instructional questioning, safety, contextual conditions, and evidence of adaptations discussed during the preceding supervisory dialogue.

**Observation Domains**

| **Domain** | **Evidence categories** |
| --- | --- |
| Lesson organization and transitions | Clear structure; smooth or disrupted transitions; insufficient time |
| Student participation | Broad, uneven, or limited participation; difficult to determine |
| Grouping and differentiation | Homogeneous or heterogeneous grouping; differentiated tasks; no differentiation evident |
| Use of space and equipment | Effective or restricted space use; creative equipment use; equipment shortage |
| Task modification | Implemented as discussed; modified; not implemented; new adaptation |
| Instructional questioning | Open or closed questions; student elaboration; limited response |
| Safety | Safety evident; concern observed; not addressed |
| Discussed adaptations | Direct adoption; adapted implementation; selective non-adoption; not evident |

**Adaptation Codes**

- **Direct adoption:** The recommendation was implemented substantially as discussed.
- **Adapted implementation:** The recommendation was implemented with contextual modifications.
- **Selective non-adoption:** A recommendation or component was intentionally not implemented.
- **Not evident:** No observable evidence of the recommendation appeared during the lesson.
